# Improving the outcome of patients with castration-resistant prostate cancer through rational drug development

**DOI:** 10.1038/sj.bjc.6603223

**Published:** 2006-09-19

**Authors:** G Attard, D Sarker, A Reid, R Molife, C Parker, J S de Bono

**Affiliations:** 1 Cancer Research UK Centre for Cancer Therapeutics, Institute of Cancer Research, Royal Marsden Hospital, Sutton, Surrey SM2 5PT, UK

**Keywords:** castration resistance, prostate cancer, targeted therapies

## Abstract

Castration-resistant prostate cancer (CRPC) is now the second most common cause of male cancer-related mortality. Although docetaxel has recently been shown to extend the survival of patients with CRPC in two large randomised phase III studies, subsequent treatment options remain limited for these patients. A greater understanding of the molecular causes of castration resistance is allowing a more rational approach to the development of new drugs and many new agents are now in clinical development. Therapeutic targets include the adrenal steroid synthesis pathway, androgen receptor signalling, the epidermal growth factor receptor family, insulin growth factor-1 receptor, histone deacetylase, heat shock protein 90 and the tumour vasculature. Drugs against these targets are giving an insight into the molecular pathogenesis of this disease and promise to improve patient quality of life and survival. Finally, the recent discovery of chromosomal translocations resulting in the upregulation of one of at least 3 ETS genes (ERG, ETV1, ETV4) may lead to novel agents for the treatment of this disease.

Prostate cancer is the most common malignancy in western societies and the second most common cause of male cancer-related death in the UK (http://www.cancerresearchuk.org). Despite the significant health burden of advanced prostate cancer, the underlying biology of this disease is only now being unravelled. A wealth of new laboratory data is fuelling rational, molecularly targeted drug development programmes (Table 1) and some of the challenges specific to the clinical study of this disease are being overcome (Table 2). Furthermore, the recently described chromosomal rearrangement of ERG, ETV1 or ETV4, members of the ETS family of oncogenes, repositioning adjacent to the androgen-dependent promoter TMPRSS2 in up to 80% of prostate cancers, is the first recurrent translocation described in a common solid epithelial tumour and has been proposed to be a key process in prostate carcinogenesis (Tomlins *et al*, 2005). The prevalence of this genomic change and the potential therapeutic value of targeting its transcriptional products are as yet unconfirmed. However, the molecular biology of castration resistance is being elucidated and this is now resulting in hypothesis-testing clinical trials in patients with this disease. It is envisaged that this will have a significant impact on the limited efficacy of treatments currently available for patients with castration-resistant prostate cancer (CRPC).

## CURRENT TREATMENTS

**Figure 1:**
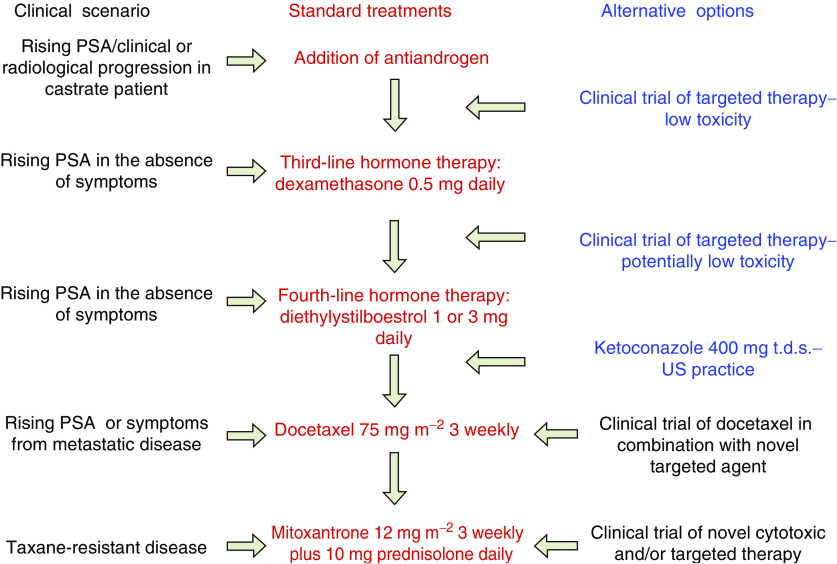
Treatment flow chart for patients diagnosed with CRPC.

Activation of the androgen receptor (AR) regulates transcription of a diverse range of target genes involved in prostate cell proliferation, differentiation and apoptosis. Drugs that reduce circulating levels of androgens or that competitively inhibit the action of androgen at the AR (antiandrogens) remain central to the treatment of prostate cancer. Gonadotrophin-releasing hormone (GnRH) agonists, such as goserelin and leuprorelin, inhibit luteinising hormone (LH) secretion and suppress testicular testosterone generation (medical castration). However, the duration of response to castration is short (12–33 months) and in almost all patients, is followed by the emergence of a castration-resistant phenotype (also referred to as hormone refractory, HRPC). Antiandrogens (e.g. bicalutamide or flutamide) can be used in combination with GnRH agonists to achieve ‘maximum androgen blockade’. However, although low levels of androgens remaining after castration are thought to play a role in the onset of CRPC, this combination strategy has not proven to prolong survival. In addition, up to 30% of patients experience a drop in prostate specific antigen (PSA) after discontinuing antiandrogens (Small *et al*, 2004), explained in part by the development of AR gene mutations, or gene amplification and increased AR expression that may cause AR antagonists to behave as agonists (Chen *et al*, 2004). Continuous low dose, oral glucocorticoids can also result in temporary PSA responses in approximately 25% of patients, presumably due to adrenal androgen suppression (Berthold *et al*, 2005) (Figure 1). High doses of oestrogens can also modulate AR function and clinical studies have confirmed antitumour activity in CRPC. However, as a result of their unfavourable toxicity profile, oestrogens are generally deferred to fourth-line hormonal therapy (Figure 1). Diethylstilboestrol (DES) is currently being investigated in combination with docetaxel after the observation that it suppresses taxane-resistant *β*-tubulin isotypes. *In vitro,* this has been shown to accentuate G2/M arrest in prostate cancer cell lines by an oestrogen-receptor independent mechanism, thus improving the therapeutic efficacy of docetaxel (Montgomery *et al*, 2005).

Treatment with docetaxel 75 mg m^−2^ and daily 10 mg prednisolone has been shown to confer a survival advantage of a median of 2.5 months and improve quality of life in patients with CRPC when compared to mitoxantrone and is now regarded as the standard of care in patients for whom chemotherapy is indicated (Tannock *et al*, 2004). Platinum containing regimens such as epirubicin, carboplatin and 5-flurouracil have also shown antitumour activity in phase II studies (Birtle *et al*, 2004) and their activity in patients who fail docetaxel treatment is undergoing evaluation. Satraplatin (JM-216), an oral platinum compound, is currently being evaluated in a large international trial (SPARC), in combination with prednisolone as second-line treatment after docetaxel. Novel tubulin binding drugs such as the epothilone B analogue, ixabepilone (BMS-247550)(Galsky *et al*, 2005) and the halichondrin B analogue, E7389 (Kuznetsov *et al*, 2004), have demonstrated promise in the post-docetaxel setting and clinical trials are ongoing.

The major impact on the quality of life of most patients with CRPC occurs as a result of their disease metastasising to bone. Single fraction palliative radiotherapy can be used to control pain from solitary sites of disease. The use of bisphosphonates, such as zoledronic acid, remains controversial and although one study suggests a reduction in skeletal-related complications, overall no improvement in patient quality of life or survival has been reported (Saad *et al*, 2004). For patients with widespread painful bone involvement, treatment with bone-seeking radiopharmaceuticals, for example strontium-89, is available in specialist centres and offers a targeted approach for providing rapid pain relief. The less myelosuppressive radioisotope, the *α*-emitter radium 223, is also currently in phase II studies, and may be superior to strontium as administration of higher doses is not as limited by marrow toxicity (Nilsson *et al*, 2005).

## WHY CURRENT THERAPIES FAIL: MOLECULAR MECHANISMS CAUSING CASTRATION RESISTANCE

**Figure 2:**
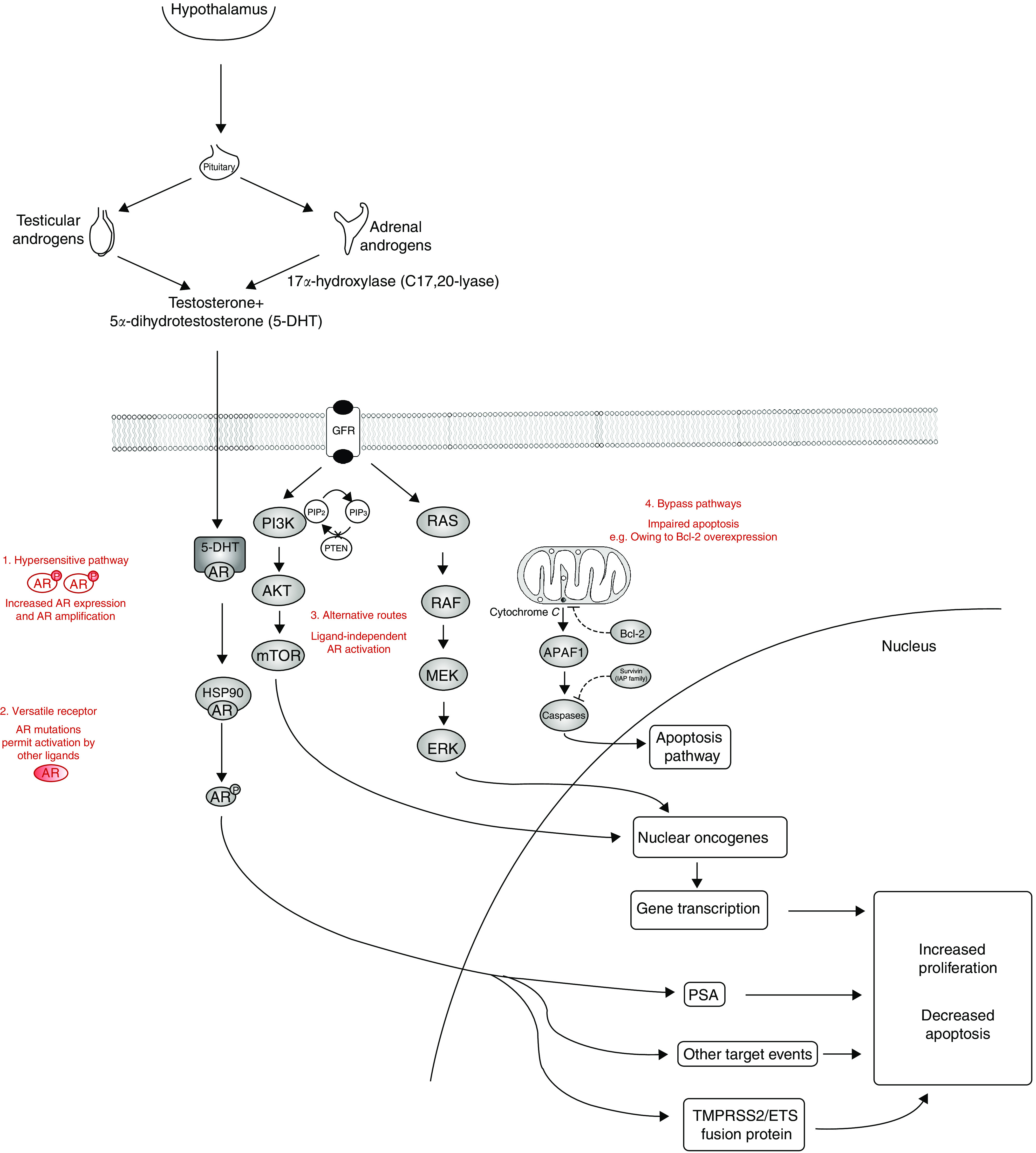
Novel therapeutic targets and proposed mechanisms of androgen resistance (text in red) in hormone refractory prostate cancer. (1) Hypersensitive Pathway: AR amplification, increased AR expression or alterations in corepressor/coactivator function. (2) Versatile receptor: mutations in the ligand-binding domain of the AR permitting nonandrogenic ligand binding. (3) Alternative routes: utilisation of AR machinery by alternative pathways, that is, PI3K/Akt. (4) Bypass pathways: bypassing of the AR and its cellular machinery entirely, that is, upregulation of the antiapoptotic protein Bcl-2. Therapeutic targets include: the adrenal steroid synthesis pathway, AR signalling, growth factor receptors (GFR), PTEN (phosphatase and tensin homolog) and PI3K (phosphatidylinositide 3-OH kinase) signalling, angiogenesis and apoptosis. HSP90 denotes heat shock protein 90; PIP2 phosphatidylinositol-4,5- bisphosphate; PIP3 phosphatidylinositol-3,4,5- triphosphate; Akt protein kinase B; mTOR mammalian target of rapamycin; oncogenes Ras, Raf, Mek, Erk; Bcl-2 antiapoptotic protein; APAF1 apoptotic peptidase activating factor 1; IAP inhibitor of apoptosis family (of which survivin is a member). Solid lines indicate promotion. Broken lines indicate inhibition.

The restricted therapeutic options available to prostate cancer patients when hormonal therapy fails underscores the urgent need for the development of novel agents to tackle the castration refractory state. The key to drug development lies in identifying and characterising targets and pathways driving cancer growth in this disease. As prostate cancer progresses, prostate cancer cells evolve, developing mechanisms to survive in an androgen-depleted environment. Postulated and documented resistance mechanisms are discussed below and in Figure 2.

### Altered AR sensitivity, amplifications and mutations

Continued (androgen-dependent) PSA secretion and the presence in tumour samples of androgens and AR mRNA expression at levels associated with active AR signalling strongly suggest that reactivation of the AR and AR-responsive pathways is one mechanism by which tumours become resistant to androgen deprivation (Titus *et al*, 2005). The peripheral conversion of adrenal androgenic steroids (primarily androstenedione) to testosterone by 17-ketoreductase could account for these intratumoural androgens although it has been hypothesised that altered regulation of tumour enzymes involved in the synthesis and inactivation of androgens may be one cause for their accumulation.

Prostate cancer cells also circumvent the effects of androgen blockade, by developing the ability to use very low levels of androgen to grow. DNA amplification resulting in increased AR expression can result in a receptor capable of activation with low levels of ligand, further supporting AR signalling as a mechanism for castration resistance. In isogenic cell lines, increased expression of AR mRNA by less than two-fold results in resistance to antiandrogens (Chen *et al*, 2004).

There is therefore increasing evidence that a role may exist for novel strategies to target the AR and inhibit androgen synthesis, with the aim of creating an androgen-free environment in prostate tumours. Alternative therapeutic targets relating to the AR include heat shock proteins (HSP) and histone deacetylases (HDAC) whose role is in maintaining the function and stability of the AR.

### AR gene mutations and altered ligand specificity

While androgens are pivotal for the growth of CRPC, mutations in the AR lead to its activation by nonandrogenic steroid molecules and antiandrogens (Figure 2). The majority of AR mutations found in this disease are located at the ligand-binding domain. These occur more frequently in CRPC, and may, in part, explain why 10–30% of patients receiving antiandrogens experience a paradoxical PSA drop on cessation of treatment (Small *et al*, 2004).

### Downstream signalling of the AR

A number of signal transduction processes have been implicated in activation of downstream AR signalling that is independent of ligand binding to the AR. These include the epidermal growth factor receptor family, the insulin growth factor-1 receptor, and the phosphoinositide 3-kinase pathway (Figure 2).

### Bypass pathways

One of the most important mechanisms in the development of CRPC is the induction of a bypass pathway independent of the AR that can overcome apoptosis induced by androgen depletion. By bypassing the AR completely, prostate cancer cells survive independent of ligand-mediated or nonligand-mediated AR activation (Figure 2). One such example of this is the upregulation of antiapoptotic proteins, including bcl-2 or survivin.

### Stem cells

Prostate cancer stem cells (PCSC) are rare, undifferentiated cells that do not express the AR and are not dependent on androgens for survival. Prostate cancer stem cells are thought to be responsible for maintaining the tumour and as they are able to survive androgen deprivation therapy (ADT), they could result in relapse with castration-resistant disease several months to years after an initial response. PCSC have been isolated from tumour samples based on high surface expression of integrin *α*_2_*β*_1_ and CD133 and are being screened for unique characteristics that will allow their selective targeting with novel therapeutic agents (Collins *et al*, 2005).

## OPTIMISING THE SUCCESSFUL DEVELOPMENT OF AGENTS FOR CRPC

With our increasing understanding of the genes and pathways that drive CRPC, therapeutic agents can be developed that act on the molecular targets defined by the key genetic and molecular abnormalities responsible. With a plethora of new agents becoming increasingly available for CRPC, it is critical that the drug development process is optimised to ensure the cost and length of time required for the approval of successful drugs is reduced. Critical to this process is the use of pharmacokinetic (PK) and pharmacodynamic (PD) end points, allowing a pharmacological ‘audit trail’ to be constructed. PK end points provide information on how much drug enters the body, and ideally into target tissues; PD end points allow understanding of what the drug does with respect to modulation of the molecular target and biochemical pathway, and whether this translates into achieving the desired biological effect (e.g. apoptosis). This ensures that all of the key stages in prostate cancer drug development – from preclinical models testing and drug administration in patients, through to the biological effect and the clinical outcome – can be monitored and interpreted (Workman, 2003). The audit trail thus provides a basis for answering critical questions in a rational, hypothesis-driven fashion and ensures that the drug development process is scientific and rigorous. In CRPC this is complicated by the inaccessibility of primary and metastatic tumour (solely bone metastasis in most patients), and by the lack of technology for assessing response to drugs (no measurable visceral disease by CT/MRI, and inadequacy of PSA assay) (see Table 2 and Ongoing Challenges).

## TARGETING AR SIGNALLING

### Inhibition of the steroid synthesis pathway

Ketoconazole, an azole antifungal agent that weakly inhibits several cytochrome P450 enzymes involved in adrenal steroid synthesis has been utilised to treat CRPC. Twenty to 35% of patients who progress on antiandrogens have a short-lived PSA response to ketoconazole and on progression postresponse have a significant rise in plasma adrenal androgens (Small *et al*, 2004) suggesting that this may be related to ketoconazole resistance.

A more specific inhibitor of androgen synthesis is currently in clinical development. Abiraterone acetate (AA) is a potent, orally bioavailable and irreversible inhibitor of 17*α*-hydroxylase/C17, 20-lyase: a key enzyme involved in the 17-hydroxylation of pregnenolone and progesterone and their subsequent conversion to the adrenal androgens, dehydroepiandrosterone (DHEA) and androstenedione. Its inhibition causes a reduction in testosterone levels (O’Donnell *et al*, 2004). A series of three phase I trials investigating the ability of AA to cause maximum suppression of testosterone synthesis when given to castrate and noncastrate males with prostate cancer has been conducted (O’Donnell *et al*, 2004). Abiraterone acetate, administered as a single dose to castrate males, achieved significant further suppression of testosterone and androstenedione that lasted from 2 to 5 days. In these phase I studies, AA was well tolerated and although there was no clinical or biochemical evidence of glucocorticoid deficiency, a reduced cortisol response to ACTH stimulation after 12 days continuous AA dosing was reported, in keeping with a reduction in adrenocortical reserve. Continuous, daily AA is currently being studied in castrate CRPC patients who have failed GnRH agonists. Although sufferers of familial 17*α*-hydroxylase deficiency do not develop adrenocortical deficiency, patients on continuous AA may require glucocorticoid rescue. Drugs targeting steroidogenesis, the aromatase inhibitors, have been successfully used in the treatment of breast cancer and AA may impact the management of CRPC in a similar fashion.

### Ablation of intracellular AR signalling

Heat shock protein 90 is a member of the family of heat shock proteins that acts as an ATP-ase driven molecular chaperone. Heat shock proteins90 is required for the stability, conformation, function and regulation of a number of key oncogenic ‘client proteins’, including ErbB2 and AKT, as well as several transcription factors including AR (Chen *et al*, 2005). In prostate cancer animal models, the first-in-class HSP90 inhibitor 17-allylaminogeldanamycin (17-AAG) causes the degradation of these client proteins at nontoxic doses and inhibits the growth of hormone-naive and castration-resistant tumours (Solit *et al*, 2003). 17-allylaminogeldanamycin induced cell-cycle arrest and apotosis *in vitro* and *in vivo*, and phase I trials have now been completed (Banerji *et al*, 2005). These studies provided the proof-of-concept for HSP90 inhibition at tolerable doses, as measured by changes in client protein and HSP expression in peripheral blood lymphocytes as well as tumour tissue of treated patients.

Histone deacetylase inhibitors can also inactivate the HSP90 chaperone and hence can deplete key client proteins such as AR. LAQ824 and SAHA have demonstrated activity in preclinical models, with depletion of AR through HSP90 inactivation (Chen *et al*, 2005). Recently, aberrations of ‘global’ histone modification have been observed in prostate cancer. Global levels of the acetylation or methylation of five different residues in histones H3 and H4 were examined in prostate tumour samples; these histone modification patterns can predict tumour grade and recurrence (Seligson *et al*, 2005). Phase II trials of HDAC inhibitors are now underway in CRPC, and interim results of FK228, a depsipeptide that inhibits HDAC, were presented at ASCO 2006 by Molife *et al* (abstract 217) and suggest clinically relevant antitumour activity.

## TARGETING THE VITAMIN D RECEPTOR

Calcitriol (1,25-dihydroxycholecalciferol), the principal active metabolite of vitamin D, demonstrates significant antineoplastic activity in preclinical models of prostate cancer. The vitamin D receptor, a member of the nuclear steroid hormone receptor superfamily, mediates transcriptional activation of cyclin-dependent kinase inhibitors causing G0/G1 cell-cycle arrest. Calcitriol also modulates growth factor signalling, induces apoptosis through downregulation of the antiapoptotic protein Bcl-2 and is antiangiogenic (Beer *et al*, 2005). The concentration of calcitriol required for antineoplastic activity *in vitro* far exceeds the normal physiologic range and daily dosing is not achievable owing to hypercalcaemia and hypercalcuria. Intermittent oral administration of calcitriol has been established as a means of achieving potentially therapeutic peak concentrations. Interim results from the double-blind randomised trial of docetaxel with or without calcitriol (DN-101) (ASCENT) were presented at ASCO 2005 by Beer *et al* (Abstract 4516). Patients received weekly docetaxel for 3 weeks of a 4-week cycle and either oral DN-101 or placebo. At a median of 18 months follow-up, survival data from 250 patients favours DN-101 (23.5 months) over placebo (16.4 months) (*P*=0.035; HR=0.67). Low calcaemic vitamin D analogues, such as seocalcitol (EB1089), have demonstrated antiproliferative effects in preclinical models and may allow improved dose delivery and antitumour activity.

## TARGETING SIGNALLING IN CRPC

The development of agents to target specific upstream genes and proteins potentially deregulated in prostate cancer also provides an opportunity for anticancer therapy for CRPC. A number of potential therapeutic targets have been elucidated for CRPC. The most common genetic alteration in CRPC is probably loss of the *PTEN* tumour-suppressor gene. In addition, profiling studies have implicated a number of receptor tyrosine kinases as being overexpressed in differing stages of prostate cancer, including the erbB kinase family (ErbB1 (epidermal growth factor receptor, (EGFR)), ErbB2 (HER2/neu), and ErbB3 (HER3)), the insulin-like growth factor receptor (IGF-1R), and the platelet-derived growth factor receptor (PDGF-R).

## PTEN AND PHOSPHOINOSITIDE 3-KINASE SIGNALLING

The phosphoinositide 3-kinase (PI3K) pathway regulates many key cellular processes. There is now overwhelming evidence implicating the PI3K/AKT/mTOR pathway as a regulator in the malignant progression of prostate cancer. Functional loss of PTEN (which is the negative regulator of PI3K) is thought to occur in up to half of all prostate cancers, and is associated with increased activation of AKT and the downstream kinase mTOR, which is involved in regulating protein synthesis. Loss of PTEN and increased AKT-1 phosphorylation is typically associated with higher Gleason grading, advanced stage and poorer prognosis (Ayala *et al*, 2004). The PI3K pathway appears to be critical in the development of CRPC: *in vitro* data suggest that overexpression and activation of AKT can trigger prostate cancer androgen escape via altered sensitivity and activation of AR (Edwards and Bartlett, 2005).

The PI3K pathway therefore presents a number of attractive kinase targets for drug development. The first generation of PI3K inhibitors were limited by lack of potency, poor selectivity for the oncogenic class I PI3K isoforms, and unsuitable pharmaceutical properties. Newer generation inhibitors have improved pharmacologic properties, appear highly selective and have demonstrated growth inhibition *in vitro* and *in vivo*; a number of these inhibitors are shortly to enter the clinic (Hennessy *et al*, 2005). Preliminary data from these inhibitors show that the likely molecular response is G1/S phase arrest, with no significant apoptosis; PI3K inhibitors may therefore be best used in combination with inhibitors of other survival signalling pathways, for example, EGFR/MEK/MAPK (She *et al*, 2005) or following treatment with cytotoxics. Similarly, a number of specific small molecule inhibitors of AKT are in development and should enter clinical trials shortly. Proof of principle that the PI3K pathway can be successfully targeted for clinical use in cancer has been demonstrated by the development of rapamycin analogues (CCI-779, RAD-001) that inhibit the mTOR kinase and are now undergoing evaluation in phase II trials in CRPC. RAD-001 has been shown to completely reverse the prostate intraepithelial neoplasia (PIN) phenotype in murine transgenic AKT models; in addition mTOR-dependent regulation of HIF-1*α* may produce an antiangiogenic effect (Majumder *et al*, 2004). One of the main concerns regarding the use of mTOR inhibitors is the possibility of ‘negative feedback’ with activation of upstream targets such as IGF-1R and p-AKT (O’Reilly *et al*, 2006).

### IGF-1R signalling

Activation of IGF-1R by IGF-1 or IGF-2 results in phosphorylation and membrane recruitment of insulin receptor substrate (IRS) proteins and activation of intracellular signalling pathways, including PI3K and MAPK. The activated receptor is thus able to induce AR signalling in the absence of AR ligand activation. IGF-1R expression has been reported to alter as prostate cells progress from a normal to a malignant phenotype and IGF-1R is implicated in resistance to therapy (Pollak *et al*, 2004). Targeting of IGF-1R signalling in preclinical tumour models has suppressed growth of prostate cancer cells, induced apoptosis *in vitro* and *in vivo* and has sensitised cancer cells to conventional chemotherapeutic treatment and irradiation (Burtrum *et al*, 2003). An IGF-1R targeting monoclonal antibody is now being evaluated for the treatment of CRPC.

### ErbB receptors

A number of studies suggest that crosstalk between the activated ErbB receptor kinase axis and the AR receptor signalling pathway may be important for the growth and survival of both hormone-sensitive and CRPC. Epidermal growth factor receptor expression correlates with Gleason score and disease progression and has been implicated in the progression to castration resistance (Di Lorenzo *et al*, 2002). Signalling mediated through ErbB2/ErbB3 is implicated in AR activation through effects on AR DNA binding and stability (Mellinghoff *et al*, 2004). However, phase II studies of single agent ErbB signalling pathway inhibitors, including gefitinib (a small molecule EGFR tyrosine kinase inhibitor), trastuzumab (a monoclonal antibody to HER2) and pertuzumab (a HER2 dimerisation inhibitor) have reported no clinically significant activity in unselected CRPC patients (Ziada *et al*, 2004; Canil *et al*, 2005; de Bono *et al*, 2005).

## OTHER STRATEGIES

One of the important mechanisms in the development of CRPC is the induction of a bypass pathway independent of AR that can overcome apoptosis induced by androgen depletion. Survivin is a member of the inhibitor of apoptosis (IAP) family and is a candidate gene that can block apoptosis. Survivin has been associated with phenotypically aggressive prostate carcinoma and with resistance to antiandrogen therapy (Zhang *et al*, 2005). Small molecule inhibitors of survivin function have demonstrated efficacy in preclinical prostate models, and are in phase I/II clinical trials. Bcl-2 is another apoptosis inhibiting protein, and its expression in CRPC correlates with a worse prognosis. In prostate cancer models, Bcl-2 antisense oligonucleotides inhibited the expression of Bcl-2, delayed the development of androgen independence and enhanced the effects of chemotherapy. A phase II study of the Bcl-2 antisense G3139 (oblimersen sodium) and docetaxel has shown promising antitumour activity and phase III trials are now ongoing (Tolcher *et al*, 2005).

An alternative strategy is targeting neovascularisation. Progression of prostate cancer has been associated with increased angiogenesis, at least in part associated with upregulation of proangiogenic factors, such as members of the VEGF family (Li *et al*, 2005). Antiangiogenic agents can reduce intratumoural interstitial pressure and increase anticancer drug delivery and enhance radiotherapy efficacy (Jain R, *Science* 2005). Bevacizumab, a humanised murine monoclonal antibody to VEGF, has resulted in clinical benefit in a number of tumour types, including colorectal, non-small-cell lung and breast cancer, and is currently being evaluated in CRPC in a randomised double-blinded, placebo-controlled phase III study administering docetaxel with or without bevacizumab (CALGB 90401).

Endothelin-1, via the endothelin-A receptor, inhibits apoptosis, stimulates proliferation of prostate cancer cells and osteoblasts and induces neovascularisation in response to hypoxia, making this effector pathway a promising therapeutic target. Atrasentan, a selective oral ET-A receptor antagonist, has been tested in patients with CRPC but its antitumour activity has been insufficient to warrant regulatory approval for its inclusion in the treatment of advanced CRPC with bone metastasis (Carducci *et al*, 2003). Phase III data from the treatment of patients with earlier stage disease treated with atrasentan is awaited.

## ONGOING CHALLENGES

It is envisioned that the next decade will result in significant changes in the treatment of prostate cancer (Table 2). A key challenge that remains to be addressed is the identification of suitable surrogate end points for survival. Prostate specific antigen does not predict survival and therefore, is not suitable for evaluating response to drugs. Recently, the rate of rise of PSA (PSA velocity) has been associated with the length of survival after treatment and may prove to be a suitable surrogate for survival (Rozhansky *et al*, 2006). However, PSA secretion is driven by AR signalling and agents that directly target AR signalling could be ineffective treatments yet modulate PSA levels. New technology allows the isolation and enumeration of circulating tumour cells (CTC) in patients with advanced prostate cancer. The number of CTC pre- and post-chemotherapy above and below a set threshold has proved to be a true surrogate marker for response and survival in advanced breast cancer (Cristofanilli *et al*, 2004) and trials are currently ongoing investigating this technology in prostate cancer. Diffusion-weighted magnetic resonance imaging and positron emission scans using ^18^F-fluoro-2-deoxy-D-glucose or FLT ^18^F-fluoro-3′deoxy-3′-L-fluorothymidine may also be valuable tools to assess CRPC and may allow early assessment of disease response. These surrogates could become primary end points in efficacy trials expediting future advances and drug approval.

## Figures and Tables

**Table 1:** Factors specific to advanced prostate cancer that make the development of new treatments challenging

**Challenge**	**Solution**
1. Preclinical models are not representative of clinical behaviour of disease	Generation of new cell lines and novel tumour models, particularly models with TMPRSS2/ETS gene translocations
2. Metastatic castration resistant tissue is difficult to access (many patients have solely bone or inaccessible intra-abdominal metastasis)	Recruitment of patients to research projects involving tumour tissue acquisition
3. Biology underlying CRPC is not well defined	Increased funding of research in CRPC and interest among the scientific community and translational researchers
4. No measurable disease in most patients and inadequate surrogates of clinical benefit limit utility of small nonrandomised studies	Evaluation and establishment of new surrogate markers, for example, enumeration of circulating tumour cells, PET imaging

CRPC, castration resistant prostate cancer; PET, positron emission tomography.

**Table 2:** New drugs under investigation for the treatment of CRPC

**Target**	**Biological effect**	**Drug**	**Stage of clinical development**
17*α* hydroxylase/C17, 20 lyase	Suppression of adrenal androgen precursors	Abiraterone acetate	Phase I/II
HSP90	Inhibition of AR signalling	17-AAG	Phase II
		17-DMAG	Phase I
HDAC	Downregulation of AR	SAHA	Phase II
		FK228	Phase II
Vitamin D receptor	Agonism of VDR antiproliferative effects	DN-101	Phase III
		EB1089	Phase I
PI3 kinase	Inhibit PI3K signalling axis	P1-103 ZSTK474	Phase I trials anticipated to start in 2006
mTOR	Inhibition of mTOR-dependent protein translation	CCI-779	Phase II
		RAD001	Phase II
IGF1-R	Inhibit IGF1-R signalling axis	CP-751, 851	Phase I
			Phase II trials anticipated to start 2006
ErbB receptor family	Inhibit erbB signalling axis	Gefitinib	Negative phase II trials
		Pertuzumab (2C4)	Negative phase II trials
Survivin	Proapoptotic	YM-155	Phase II
BCl-2	Proapoptotic	G3139	Phase II
VEGF	Antiangiogenesis	Bevacizumab	Phase III
VEGFR	Antiangiogenesis	BAY 43-9006	Phase II
		AZD2171	Phase I/II
ET_A_	Inhibition of endothelin-1 axis	Atrasentan	Phase III trials (first trial did not meet its end point)
*β*-Tubulin	Cell-cycle arrest	Ixabepilone	Phase II/Phase III
		E7389	

HSP, heat shock protein; AR, androgen receptor; 17-AAG, 17- allylamino-17-demethoxygeldanamycin; 17-DMAG, 17-dimethylaminoethylamino-17-demethoxygeldanamycin; HDAC, histone deacetylase; SAHA, suberoylanilide hydroxyamic acid; PI3K, phosphatidylinositol-3-kinase; mTOR, mammalian target of rapamycin; IGF1-R, insulin growth factor receptor; VEGF (R), vascular endothelial growth factor (receptor); ETA, endothelin-A.
